# SRSF2 mutations in myelodysplasia/myeloproliferative neoplasms

**DOI:** 10.1186/s40364-018-0142-y

**Published:** 2018-09-26

**Authors:** Amandeep Aujla, Katherine Linder, Chaitanya Iragavarapu, Michael Karass, Delong Liu

**Affiliations:** 1 Department of Medicine, New York Medical College and Westchester Medical Center, Valhalla, NY USA; 2 Section of Hematology-Oncology, Department of Medicine, Baylor College of Medicine, Houston, TX USA; 3 Division of Blood and Marrow Transplantation, Department of Medicine, Stanford University, Stanford, CA USA; 4 The affiliated Cancer Hospital of Zhengzhou University and Henan Cancer Hospital, 127 Dongming Road, Zhengzhou, 450008 China

## Abstract

Recurrent gene mutations have been described with varying frequencies in myelodysplasia (MDS) /myeloproliferative neoplasm (MPN) overlap syndromes (MMOS). Recent work has placed significant focus on understanding the role of gene lesions involving the spliceosomal machinery in leukemogeneis. SRSF2 is a gene encoding critical spliceosomal proteins. SRSF2 mutations appear to play an important role in pathogenesis of MMOS, particularly in chronic myelomonocytic leukemia. Inhibition of splicing may be a new therapeutic approach. E7107, a spliceosome inhibitor, has been shown to differentially inhibit splicing more in SRSF2-mutant cells leading to decreased leukemia burden in mice. H3B-8800 is a small molecule modulator of spliceosome complex and has been shown to lower leukemia burden in SRSF2-P95H mutant mice. This review focuses on the incidence of mutant SRSF2 across various MMOS as well as recent clinical development of spliceosome inhibitors.

## Background

Myelodysplastic and myeloproliferative overlap syndromes (MMOS) were initially recognized as a unique entity in the third edition of WHO classification of myeloid neoplasms [1]. This group initially had three disorders - chronic myelomonocytic leukemia (CMML), atypical chronic myeloid leukemia, BCR-ABL1^−^ (aCML), and juvenile myelomonocytic leukemia (JMML). The fourth entity, MDS/MPN with ringed sideroblasts and thrombocytosis (MDS/MPN-RS-T, previously known as RARS-T), was added in the 2016 revision of WHO classification [2]. Currently, MMOS also includes a fifth group, MDS/MPN unclassifiable, which is inclusive of all other MDS/MPN -like syndromes that do not meet diagnostic criteria for the above.

With the increasing use of next-gene sequencing and molecular studies in clinical practice, new patterns of gene mutations are being reported in myeloid neoplasms [3–8]. These mutations are being used as biomarkers for classification and druggable targets [9–12]. A variety of small molecules including ruxolitinib, enasidenib, midostaurin, and AG-120 are in clinical applications and/or late-stage clinical development [13–21].

MDS/MPN overlap syndromes can present with overlapping clinical and morphological features of both MDS (peripheral cytopenia and/or dysplastic bone marrow) and clonal proliferation (leukocytosis, thrombocytosis or organomegaly) during the initial diagnosis [22]. Genomic aberrations have been reported at a frequency as high as 75% along with multiple somatic mutations [23]. Most common mutations reported are TET2, ASXL1 and/or SRSF2 in CMML, NRAS/KRAS in JMML, SETBP1 in aCML and JAK-STAT and/or SF3B1 in MDS/MPN-RS-T [24–27]. This review focuses on SRSF2 mutations across various entities of MMOS.

## SRSF2

SRSF2 (Serine and arginine Rich Splicing Factor 2), also called SC35 and SRp30b, belongs to the SR (Serine and Arginine rich) protein family [28, 29]. It was recognized first in 1990 by Fu and Maniatis using a monocloncal antibody developed against mammalian spliceosomes [30]. It was reported to play a role in splicing during spliceosome assembly [31, 32]. SRSF2 has a RNA recognition motif and thus promotes spliceosome assembly at adjacent splice sites to allow appropriate exon inclusion [28, 33, 34]. In addition, SRSF2 was reported to play an active role in transcription elongation and in coupling transcription and splicing processes [35, 36].

## SRSF2 in oncogenesis

The oncogenic potential of SRSF2 was first demonstrated in SRSF2 knock-out mouse embryo fibroblasts (MEFs). SRSF2 mutation increased double-strand DNA breaks, p53 hyperphosphorylation and hyperacetylation with cell cycle arrest [37]. Similar findings were also duplicated in mouse hematopoietic cells, with growth arrest, early senescence and apoptosis in SRSF2 deleted cells [38]. In another study based on similar intervention, SRSF2 homozygous knockout mice showed 70–90% loss of thymocytes with significantly increased CD4-/CD8- T cells and decreased CD4+/CD8+ T cells. Thus, loss of SRSF2 seemed to affect T cell maturation in thymus, possibly secondary to altered splicing of CD45 as reported in the study [39].

While loss of SRSF2 led to decreased survival, mutant SRSF2 (SRSF2-mut) expression was associated with oncogenesis. Direct association of SRSF2 in development of myelodysplasia was demonstrated in SRSF2-P95H mutant mice [40]. P95H is the most common mutation site in the SRSF2 gene [41–45] and its proximity to RRM site of SRSF2 might play a role in altering RNA binding abilities [38] [46]. Heterozygous P95H mutant and homozygous SRSF2 deleted bone marrow mononuclear cells led to development of significant leukopenia and anemia in lethally irradiated recipient mice. However, only P95H mutated mice developed macrocytic RBCs and had normal bone marrow cellularity in contrast to bone marrow aplasia seen with homozygous SRSF2 deletion. Peripheral erythroid and myeloid dysplasia was also seen only with P95H mutant mice [40]. These findings correlate with MDS findings in humans.

SRSF2 mutant cells have been shown to require wild-type (WT) SRSF2 allele for the cell survival, explaining the phenotypic differences between heterozygous and homozygous genotypes [47]. Hemizygous SRSF2P95H/−mice had shorter survival with severe bone marrow aplasia in contrast to SRSF2P95H/+ mice (*p* = 0.004). Hemizygous cells also showed two-fold higher mis-splicing events compared to heterozygous cells [48]. Similar oncogenic associations secondary to mis-splicing have also been reported with other splicing factor mutations such as U2AF1 and SF3B1 [49] [50, 51]. SRSF2 mutation frequently occurs in close association with these and other mutations [26, 42, 44]. During disease progression of MDS, additional mutations are acquired [52].

## SRSF2 in MDS/MPN overlap syndromes

### CMML

In 2011, Yoshida et al. identified frequently recurring splicing factor mutations in a cohort of adult patients with myeloid neoplasms through performing whole-exome sequencing. SF3B1 (36%) was the most common mutation followed by SRSF2 (25.6%), U2AF35 (16.9%) and ZRSR2 (10.5%) [8]. These mutations were more frequent and comparatively more specific to the diseases with myelodysplastic features (MDS, CMML, t-AML and AML-MRC) [43]. SRSF2 mutation was reported with a high frequency of 28–47% [41–43, 53] in other cohorts of CMML patients and was reported to be significantly associated with higher age, higher hemoglobin, normal karyotype and TET2 mutation [26, 45, 54]. Interestingly, it occurred mutually exclusively with EZH2 mutation [42, 44]. No significant association had been reported with leukocytosis, blast percentage, WHO histologic categories (CMML-1 and CMML-2) or cytogenetic risk categories. No specific morphological or immunohistochemical features in the bone marrow (e.g. dysplasia, CD14 and CD34 positive cells) were significantly associated with SRSF2 mutations in a study done on MDS/MPN entities. SRSF2 testing on bone marrow specimens was shown to be 44.4% sensitive and 88.1% specific in diagnosing CMML over MDS or MPN; with modest positive likelihood ratio of 3.73 [45].

SRSF2 has been associated with worse survival outcomes in low-risk MDS patients and PMF [43, 44, 55] but evidence has not been very clear among MDS/MPN overlap syndromes. Earlier studies investigating SRSF2 mutations in MDS/MPN overlap syndromes reported no influence of SRSF2-mut on overall survival (OS) either in CMML or other MDS/MPN overlap syndromes [26, 42, 56]. The only impact SRSF2-mut had on survival was noted by Meggendorfer et al. analyzing a series of CMML patients. Patients co-harboring RUNX1 and SRSF2 mutations appeared to have improved OS compared to those who possessed wild-type (WT) variants.

An international cohort study showed poor OS outcomes associated with the SRSF2 mutations in CMML patients aged ≤65 years but with non-significant difference among leukemic transformation rates [57]. Similar outcomes with decreased OS along with decreased progression free survival (PFS) or leukemia free survival (LFS) were also reported in two studies that enrolled 56 and 312 CMML patients, respectively [27, 58]. Additional factors that negatively influenced OS as per multivariate analyses were older age (> 65 years, *p* = 0.04), WBC > 15 × 10^9^/L (*p* < 0.0001), presence of anemia (hemoglobin < 10 g/dL in women and < 11 g/dL in men; *p* = 0.0002), thrombocytopenia (< 100 × 10^9^/L; *p* < 0.0001) and an absolute lymphocyte count (ALC) > 4 × 10^9^/L (*p* = 0.03) [27, 56]. Genotypically, ASXL1 was the only mutation which predicted inferior OS and LFS in multivariate analyses.

Multiple prognostic models based on phenotypic and cytogenetic characteristics have been developed for CMML. These include the MD Anderson Prognostic Score (MDAPS) [59], the Spanish Cytogenetic Risk Stratification [60] and the CMML Prognostic Scoring System (CPSS) [61]. The MDAPS and the CPSS were dependent on clinical factors and/or basic laboratory findings. The CPSS also incorporated the Genetic Risk Score as determined by the Spanish risk stratification. More recently, attempts have been made to incorporate ASXL1 mutation status into these models based on previous data regarding impact on survival. Recently, two more prognostic models were proposed; the Groupe Francophone des Myelodysplasies (GFM) model [27] and the Mayo molecular model [62]. Of note, the GFM model was validated in a separate cohort of 165 patients with a median follow up of 27.3 months [27].

The CPSS was also updated to incorporate the impact of multiple mutations including ASXL1, NRAS, RUNX1 and SETBP1. The updated system, named CPSS-Mol, assigned variable scores to different mutations as well as the cytogenetic abnormalities [63]. A composite score was then determined using phenotypic variables as defined by CPSS and the “Genetic Risk Group”. Four risk categories were delineated and the scoring system was validated in a separate cohort of 286 patients. Lastly, prognostic implications of different types of missense mutations occurring at the P95 site of SRSF2 gene have also been reported; the P95H variant being reported to have better outcomes compared to P95L or P95A [42]. Table 1 summarizes the various studies evaluating SRSF2 mutation frequency and reported impact on overall and progression free survival in CMML.

**Table 1:** SRSF2 mutations in chronic myelomonocytic leukemia

Reference	Disease	Frequency of SRSF2 mutation	Effect on Survival	Effect on disease progression
[8]	CMML	28.4%	NR	NR
[42]	CMML	47% (129/275)	No	No
[53]	CMML	46% (173/409)	NR	NR
[41]	CMML	40% (90/226)	No	No
[43]	CMML	28%	NR	NR
[64]	CMML	20% (1/5)	NR	NR
[26]	CMML	32% (28/87)	No	NR
[77]	CMML (Chinese population)	20% (10/50)	No	No
[45]	CMML	44% (16/36)	No	NR
[57]	CMML (aged < 65 years)	45% (72/161)	Yes	No
[58]	CMML	25% (14/56)	Yes	Yes
[27]	CMML	46% (143/312)	Yes	Yes
[56]	CMML	40% (90/226)	No	No
[52]	CMML	45% (116/274	NR	No
[54]	CMML	51% (74/146)	NR	NR
[67]	CMML	53% (31/58)	NR	NR

*CMML* Chronic Myelomonocytic Leukemia, *NR* not reported

### JMML

Among a cohort of 371 children, SRSF2 mutation was only seen in 2 patients and both with normal karyotype along with co-existing RAS mutations [64]. Both patients received HSCT in the study. One relapsed with loss of SRSF2 mutation at relapse; while RAS mutation persisted. In two other studies, only 1/76 patients with JMML carried a SRSF2 mutation [26, 65]. This mutation had not been described previously and was reported as in-frame deletion in contrast to mis-sense mutations seen in adults. Although morphologically similar, genotypic characteristics of JMML are distinct from those of CMML with RAS, PTPN11, NFI and CBL. Rarity of splicing factor mutations in JMML and their loss at progression of disease likely precludes their independent role in its pathogenesis. The frequency of SRSF2 mutations in JMML was summarized in Table 2.

**Table 2:** Frequency of SRSF2 mutations in myelodysplasia/myeloproliferative neoplasms*

Studies	Disease	Frequency of SRSF2
[64]	JMML	1.7% (2/116)
[26]	JMML	0%
[65]	JMML	3.7% (1/27)
[69]	aCML	0% (0/3)
[70]	aCML	12% (3/25)
[78]	aCML	40% (24/60)
[67]	aCML	34% (12/35)
	MDS/MPN-RS-T	9% (4/45)
[71]	RARS-T	6.7% (5/75)
[72]	RARS-T	2% (1/48)
[67]	MDS/MPN-U	15% (6/39)

Abbreviations: *MDS* myelodysplasia, *MPN* myeloproliferative neoplasm, *CMML* Chronic Myelomonocytic Leukemia, *JMML* Juvenile Myelomonocytic Leukemia, *aCML* atypical Chronic Myeloid Leukemia, *MPN/MDS-U* unclassifiable MDS/MPN. * SRSF2 mutations in CMML are listed in a separate table

### Atypical CML, *BCR-ABL1*^*−*^

Atypical CML (aCML) is a rare entity among MDS/MPN overlap syndromes characterized by the absence of the BCR-ABL1 fusion gene as well as rearrangements of the PDGFRA, PDGFRB or FGFR1 genes [24]. ASXL1 (20–70%), SETBP1 (25–30%) and TET2 (43%) mutations are the most common mutations detected in aCML [66, 67]. SETBP1 mutations correlate with worse survival outcomes [68]. A high frequency of SRSF2 mutations (40%) was reported among a cohort of 60 aCML [54] while its frequency has been reported variably in other studies [67, 69, 70]. SRSF2 mutation appears more frequently with ASXL1-mut (*p* = 0.01) and SETBP1-mut (*p* = 0.004) compared to WT.

### MDS/MPN-RS-t (RARS-t)

This entity was previously known as refractory anemia with ringed sideroblasts and thrombocytosis (RARS-T) and defined under the MDS/MPN-U umbrella diagnosis. This disease has now been accepted as a separate entity in 2016 revision of WHO classification [2]. Splicing factor mutations are common in this group, with SF3B1 being the most frequently occurring mutation (85–91%) [67, 71, 72]. SF3B1 mutation status is strongly associated with increased number of ringed sideroblasts (*p* = 0.006). JAK2 (33–59%), TET2 (10–31%) and ASXL1 (20–29%) are the other frequently occurring mutations in this entity. SRSF2 is comparatively less common (2–9%) and is mostly present in association with other mutations in genotypes carrying high mutation burden [71]. In a cohort of 75 patients, SRSF2 was present in 5 cases and all of them carried ≥4 mutations [60], suggesting that it is less likely to play a driver mutation role in this entity (Table 2).

### MDS/MPN – Unclassifiable

MDS/MPN-U is another rare entity with heterogenous dysplastic and proliferative features except some distinct associations such as isolated trisomy 8 seen in about 15% of cases compared to MDS (5%) and MPN (4%) [73]. JAK2 mutation is one of the most common (23–66%) mutations reported in this group [67] but has not been reported to have prognostic importance. Other mutations occur in comparable frequencies, ASXL1 and TET2 (36%), U2AF1 (18%), SRSF2 (15%) and SF3B1 (13%). Overall, MDS/MPN-U presents a mixed picture as opposed to other MMOS. Thrombocytosis has been associated with improved survival in this group [73] but role of SRSF2 in pathogenesis or as prognostic indicator has not been well defined yet (Table 2).

## Therapeutic implications of SRSF2

Aberrant spliceosome function secondary to mutated SRSF2 has been associated with mis-splicing of multiple genes (e.g. EZH2, RUNX1, BCOR, IKAROS and CASP8, etc.) that are implicated in pathogenesis of myeloid neoplasms [40, 43]. Inhibition of splicing has been analyzed as possible therapeutic target. E7107, a spliceosome inhibitor, has shown to differentially inhibit splicing more in SRSF2-mut cells leading to decreased leukemia burden in mice [47]. A phase 1 clinical trial investigating E7107 in metastatic or locally advanced solid tumors was discontinued prematurely due to vision loss reported as adverse event in 2 cases [74]. A parallel phase I trial conducted in Europe also reported one instance of grade 4 visual disturbance secondary to optic neuritis which improved after treatment with steroids. Nevertheless, the study was discontinued for safety concerns [75].

Another compound, H3B-8800, acts as a modulator of the SF3b complex. It has demonstrated a preferential cytotoxic effect on SF3B1-mutant cells secondary to GC-rich intronic retention [76]. Decreased leukemic burden was reported in SRSF2-P95H mutant mice compared to SRSF2-WT variants. A phase I clinical trial (NCT02841540) is underway to evaluate safety of this compound in patients with MDS, AML and CMML.

## Conclusion

SRSF2 is a frequent mutation seen in MDS/MPN overlap syndromes, especially CMML. It has been shown to play a multi-faceted role during the oncogenesis of these disorders influencing transcription, splicing, translation and genomic stability. There is insufficient evidence to establish it as a primary driver mutation. Conflicting data on its prognostic role especially in CMML demand further evaluation to differentiate worse prognostic outcomes due to presence of SRSF2 mutation as opposed to other factors (e.g. presence of increased mutation burden). Targeting mis-splicing events secondary to splicing factor mutations with novel spliceosome inhibitors is an exciting approach with multiple possible therapeutic implications.

## Abbreviations

aCML: atypical Chronic Myeloid Leukemia
CMML: Chronic Myelomonocytic Leukemia
JMML: Juvenile Myelomonocytic Leukemia
MDS: myelodysplasia
MPN: myeloproliferative neoplasm
MPN/MDS-U: unclassifiable MDS/MPN
